# Lost in translation: The challenge of adapting integrated approaches for worker health and safety for low- and middle-income countries

**DOI:** 10.1371/journal.pone.0182607

**Published:** 2017-08-24

**Authors:** Glorian Sorensen, Eve M. Nagler, Pratibha Pawar, Prakash C. Gupta, Mangesh S. Pednekar, Gregory R. Wagner

**Affiliations:** 1 Center for Community-Based Research, Dana-Farber Cancer Institute, Boston, Massachusetts, United States of America; 2 Harvard T.H. Chan School of Public Health, Boston, Massachusetts, United States of America; 3 Healis, Sekhsaria Institute for Public Health, Navi Mumbai, India; Public Library of Science, FRANCE

## Abstract

**Objectives:**

To describe the process of adapting an intervention integrating occupational safety and health (OSH) and health promotion for manufacturing worksites in India and the challenges faced in implementing it; and explore how globalization trends may influence the implementation of these integrated approaches in India and other low- and middle-income countries (LMICs).

**Methods:**

This study—conducted in 22 manufacturing worksites in Mumbai, India—adapted and implemented an evidence-based intervention tested in the U.S. that integrated OSH and tobacco control. The systematic adaptation process included formative research and pilot testing, to ensure that the tested intervention was tailored to the local setting. We used qualitative methods and process evaluation to assess the extent to which this intervention was implemented, and to explore barriers to implementation.

**Results:**

While participating worksites agreed to implement this intervention, not all components of the adapted intervention were implemented fully in the 10 worksites assigned to the intervention condition. We found that the OSH infrastructure in India focused predominantly on regulatory compliance, medical screening (secondary prevention) and the treatment of injuries. We observed generally low levels of leadership support and commitment to OSH, evidenced by minimal management participation in the intervention, reluctance to discuss OSH issues with the study team or workers, and little receptivity to recommendations resulting from the industrial hygienist’s reports.

**Conclusion:**

India presents one example of a LMIC with a rising burden of non-communicable diseases and intensified exposures to both physical and organizational hazards on the job. Our experiences highlight the importance of national and global trends that shape workers’ experiences on the job and their related health outcomes. Beyond a singular focus on prevention of non-communicable diseases, coordinated national and international efforts are needed to address worker health outcomes in the context of the conditions of work that clearly shape them.

## Introduction

Working conditions shape health outcomes in complex and interrelated ways. Although work provides clear benefits, including income and opportunities to build social networks and self-esteem [1–4], exposures on the job have long been understood to contribute to occupational diseases, injuries, and fatalities [5, 6]. Job experiences, the organization of work, and workplace policies and practices may further compromise workers’ health by contributing to behaviors that are associated with risk of non-communicable diseases (NCDs)–such as tobacco use, dietary patterns associated with obesity, physical inactivity, and sleep deficiency [7, 8].

NCDs, including cardiovascular disease, cancer, diabetes, and chronic respiratory diseases, are the leading causes of death globally [9, 10], a trend also now seen in low- and middle-income countries (LMICs) [11, 12]. The chronic nature of NCDs means patients are sick longer and require more medical care, with significant cost implications [9, 10]. Growing globalization and urbanization, accompanied by changes in dietary patterns, physical inactivity, high blood pressure, and tobacco use, contribute to increases in NCD risk in LMICs [13]. The burden of these risk-related behaviors is also rising rapidly in LMICs. For example, the World Health Organization (WHO) estimates that by 2030, 80% of deaths from tobacco-related causes globally will occur in LMICs [14].

These poor health outcomes are compounded by occupational diseases and injuries in LMICs. In the last two decades, despite important economic and technological developments that have helped to reduce some occupational health problems, mainly in developed countries, exposures to occupational hazards in LMICs have generally intensified [15–17]. Globalization and new models for economic development have contributed to increasing disparities between high-income countries and LMICs. For example, working conditions have been influenced by a “race to the bottom,” whereby companies seek to enhance their competitive advantage by way of reduced wages, along with fewer worker rights and protections [16, 17]. The migration of manufacturing from higher-income countries to LMICs has also transferred hazardous exposures such as chemical substances, unshielded machinery, and unsafe materials. The emphasis on competitiveness has increased the pace of work and extended working hours [16–19]. These shifts are often accompanied by insufficient protections against hazards, the lack of enforcement of protective legislation, as well as inadequate awareness of occupational safety and health (OSH) risks. The concurrent growth in informal economies has resulted in an increasing number of workers with inadequate or no social and health protections [17, 20, 21]. Notably, evidence suggests that between 50% and 100% of workers employed in some hazardous industries in LMICs may be exposed to hazards that exceed limits set in high-income countries [15, 16].

An emerging literature has identified successful strategies to protect and promote worker health by addressing workplace hazards, the traditional domain of OSH, along with chronic disease risk, including the health behaviors associated with NCD risk. For example, the WHO developed a model to guide the implementation of healthy workplace programs that target four core avenues of influence: the physical work environment, psychosocial work environment, enterprise community involvement, and personal health resources [22]. Similar models have been proposed in a number of countries [23, 24]. In the U.S., the National Institute for Occupational Safety and Health (NIOSH) has developed its Total Worker Health^®^ initiative to provide a strategy for integrating occupational safety and health with other workplace interventions that protect and promote workers’ health, safety, and well-being, focusing particularly on the conditions of work [25]. Shared in common across this literature is the growing recognition of the importance of addressing the multiple ways in which work may influence health outcomes [26]. These integrated interventions target hazardous physical exposures on the job as well as the organizational and psychosocial forces at play as part of work, and consider the full range of health consequences for workers and their families [7, 27]. Growing evidence indicates that integrated interventions contribute to improvements in health behaviors [28–37] and employee participation in programs [38]; reduced pain, occupational injury and disability rates [39–44]; stronger health and safety programs [45, 46] and potentially reduced costs [46]. Multiple reviews have supported these findings [47–53], although a recent systematic review concluded that while integrated interventions may improve health behaviors, there is an ongoing need for further evidence on their impact on injuries and overall quality of life [54]. To date, however, the application of these approaches has focused on high-income countries, where legal and economic imperatives and a supportive normative environment in the business community are likely to encourage employer actions to protect and promote worker health [22].

India provides an important case example of the combined health impact of occupational diseases and injuries and NCDs. Approximately two-thirds of India’s population of over 1.2 billion is of working age. Although health surveillance systems are not well established, the estimated incidence of occupational diseases in India ranges between 924,700 and 1,902,300 cases per year, and there are approximately 121,000 occupational fatalities from traumatic workplace injuries per year [55]. At the same time, NCDs are the major cause of death in India, accounting for 60% of all deaths [56]. Leading risk factors for these diseases include lack of physical activity, dietary patterns associated with obesity, tobacco use and harmful use of alcohol [57]. Addressing these risk factors could contribute to a reduction in NCD-related premature deaths by 40–50% [58]. In 2010 alone, for example, an estimated 930,000 people were expected to die from conditions attributable to smoking [59]; an additional 368,000 deaths were estimated to be due to smokeless tobacco use in 2008 [60]. Forty-eight percent of men and 20% of women use some form of tobacco [61]. Use of smokeless tobacco use is high [61–63], with the result that India has one of the highest oral cancer rates in the world [64, 65], a rate that is still increasing [66]. These dual risks in LMICs takes place in the broader context of budget cuts in the public sector that contribute to reduced support for education and health, with further implications for increased health and economic inequalities [17]. Others have similarly noted that LMICs face limits in responding to the challenges of chronic diseases due to constraints across systems, including health financing, availability of health information and technologies, and limitations in the health workforce [11].

The complexity of the threats to worker health in India and other LMICs calls for sustained and effective action to better understand and address barriers to improved worker health and safety. The models referenced above have shown promise for protecting and promoting worker health in higher-income countries, but have received relatively limited attention in LMICs. There is a significant need for improved understanding of the potential applicability of these approaches for LMICs, and for adapting effective approaches to protecting and promoting worker health and safety for these low-resource settings [67]. In order to maximize the value added by research conducted in the U.S. and Europe, these strategies will need to be adapted to the unique cultural, economic, political, social and legal contexts of specific LMICs. The purpose of this paper is to describe the process of adapting an integrated worksite health protection/health promotion intervention for application in India and the challenges faced in implementing it, focusing particularly on our experiences with the OSH components. We further discuss the ways in which structural factors shaped by globalization trends may influence the implementation of integrated approaches to protecting and promoting worker health in LMICs, and offer recommendations for future applications of these approaches in other settings. We believe these experiences have implications for knowledge translation and dissemination of evidence-based approaches from high-income settings to LMICs.

## Methods

### Study overview

This paper reports our experiences in the Mumbai Worksite Tobacco Control Study, designed to assess the effectiveness of an integrated worksite tobacco control and OSH intervention called “Healthy, Safe, and Tobacco-free Worksites” [68]. This study was conducted in 22 manufacturing worksites in the Greater Mumbai region as a collaboration among the Harvard T.H. Chan School of Public Health Center for Work, Health and Well-being, the Dana-Farber Cancer Institute, the New England Research Institute in Boston, Massachusetts, and the Healis-Sekhsaria Institute for Public Health in Mumbai. This study was approved by the Healis and Harvard Chan School Institutional Review Boards, as well as by the Indian Council of Medical Research, and has been registered with ClinicalTrials.Gov (ID NCT01841879) and the Clinical Trial Registry of India. Both IRBs approved the informed consent procedure.

This cluster-randomized trial assessed differences in tobacco use quit rates between worksites randomized to receive the intervention compared to those assigned to the delayed-intervention control condition. As we have reported elsewhere, we observed a significant improvement in 30-day tobacco use cessation rates among production workers in the intervention group compared to the control (OR = 2.25, p = 0.03). The magnitude of the effect was similar for six-month sustained quit rates, although the between-group difference was not significant [68].

The *Healthy*, *Safe*, *and Tobacco-Free Worksites* intervention was designed to capitalize on existing OSH infrastructures found in Indian worksites. Here, we report on our efforts to address occupational safety and health as part of our integrated approach to tobacco control. This intervention was adapted from a series of studies testing integrated approaches to worker health in the U.S., and particularly exemplified in our WellWorks-2 Study.

### The intervention framework: A model for integrated policies, programs, and practices developed and tested in the U.S.

In studies conducted by researchers at the Center for Work, Health and Wellbeing, we have tested integrated policies, programs and practices across a range of industries, including manufacturing, health care, construction, and transportation. The WellWorks-2 study illustrates this integrated approach and provided a specific framework for adapting this intervention. This study randomly assigned 15 manufacturing worksites to a health promotion (HP) or a health promotion plus occupational safety and health intervention (HP/OSH); both groups targeted smoking and diet; the HP/OSH condition additionally incorporated reduction of adverse occupational exposures. Compared to the HP condition, the HP/OSH condition showed a significant doubling of smoking quit rates among blue-collar workers [29]; a pattern of reductions in hazardous exposures based on modifications at the source of exposure rather than based on workers’ use of personal protective equipment (PPE) [69]; and significantly greater improvements in management commitment and employee participation in OSH programs [45]. The core components of this intervention model included: (1) the use of a participatory framework by engaging workers and managers; (2) interventions to effect change in the work environment and organization, and (3) interventions for individual workers.

Joint worker–management participation: Engaging managers and workers in program planning, priority setting, and implementation is fundamental to program success [49, 70, 71]. In WellWorks-2, program committees were created to provide a channel for worker–manager input to program design and delivery. In some cases, these committees were incorporated into existing health and safety committees.

Interventions targeting the worksite organization and environment: Management was the primary channel for improving the physical work environment because management has both primary control over and responsibility for providing a safe and healthy work environment. The management intervention for OSH encouraged companies to adopt a pro-active, preventive approach, going beyond legally mandated standards for OSH to protect worker health. An industrial hygienist with the study conducted walk-through assessments of exposures and occupational health and safety programs, and provided consultations and technical assistance to management and recommended changes to reduce workers’ exposures to hazardous substances used in work processes. The intervention relied on a hierarchy of controls approach, which prioritizes changes as close to the source of exposure as possible, starting with upstream strategies for materials and process substitution, engineering controls, and as a last option, downstream interventions at the level of the worker, such as reliance on personal protective equipment (PPE) [69]. These consultations also emphasized the importance of the OSH program elements of management commitment and employee participation, hazard analysis, hazard prevention and control, and training and education [45]. We also provided consultations to support implementation of tobacco control policies and ensure the availability of healthy food options in cafeterias and vending machines, which were other core components in the work organization and environment.

Interventions for individual workers: In WellWorks-2, intervention activities were designed to address both exposure to hazardous substances and health behaviors. Using an integrated approach, program content included comprehensive, coordinated messages acknowledging the additive and sometimes synergistic effects of exposures to worksite hazards, individual health and safety behaviors, and other determinants of worker health outcomes [70]. Tobacco control programs incorporated messages concerning OSH, and OSH programs incorporated messages concerning smoking or nutrition.

### The adaptation process

To adapt this integrated approach to the Indian context, we employed a step-by-step approach to intervention development and adaptation based on principles used in our prior research in India [72] and as recommended by others. This adaptation and translation process relies on understanding the local context and setting to ensure intervention effectiveness [72, 73], and requires attention to the challenges faced in settings with low resources [74]. We conducted extensive formative research in order to assess the new setting, determine the appropriateness of the evidence-based intervention to be adapted, and examine capacity to implement the intervention based on local resources [75, 76]. We also followed prior recommendations to pilot test the adapted intervention, in order to identify barriers and facilitators to its implementation and determine training likely to be needed. We applied other recommendations to distinguish between core components, which are essential and indispensable to the effectiveness of the intervention, and adaptable elements, allowing for localizing the intervention without undermining the intervention’s integrity [77–79].

### Sample

**Fig 1:**
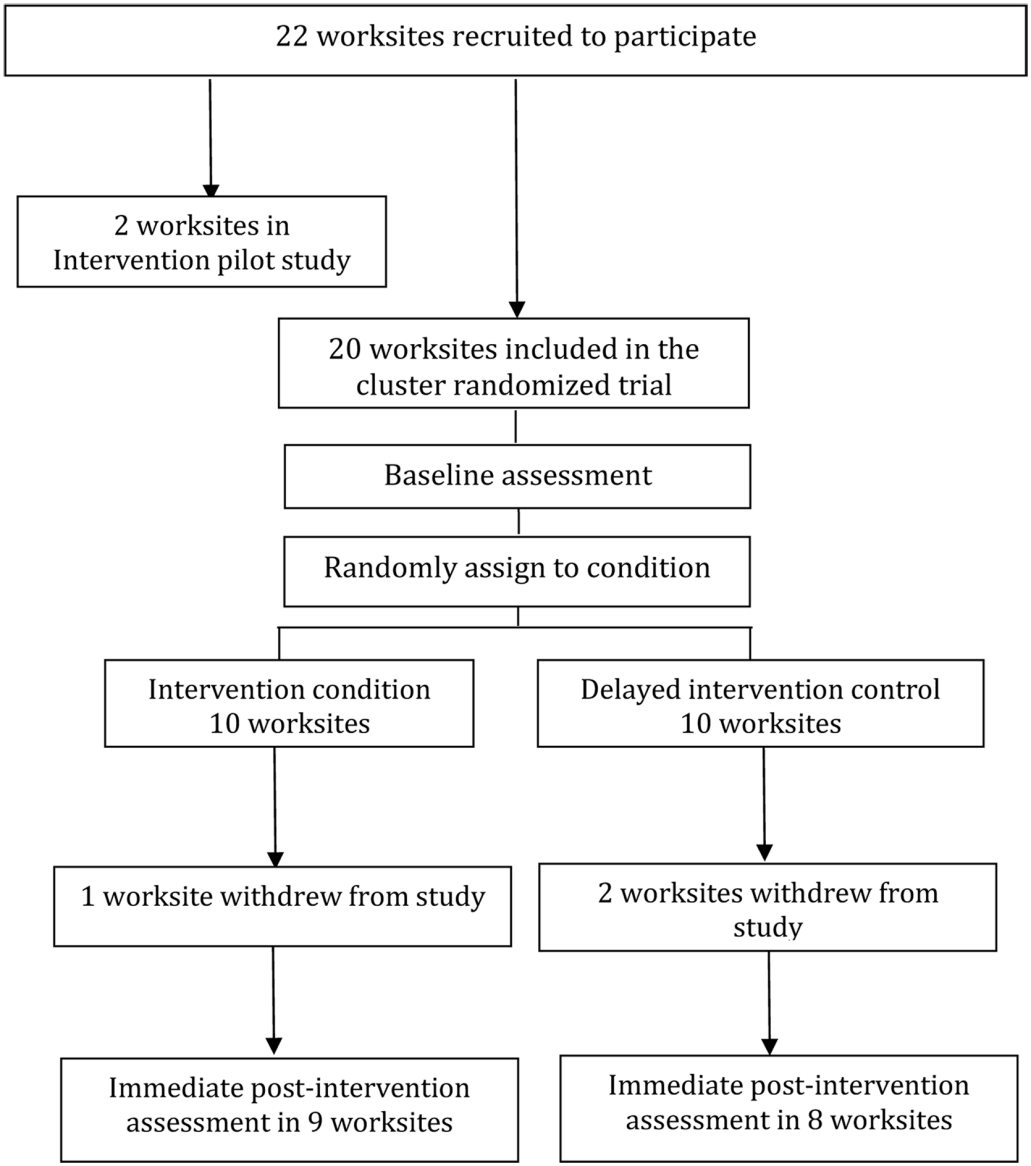
Study schema: Mumbai Worksite Tobacco Control Study.

We recruited 22 manufacturing worksites, located in the Mumbai Metropolitan Region including the Mumbai, Thane, and Raigad districts, to participate in the study [80]. Worksite eligibility criteria included: (1) size, with at least 200 production workers or at least 60% of the workforce to be comprised of production workers; (2) location in the Greater Mumbai area (i.e., Mumbai, Thane, or Raigad districts); (3) industry sector (manufacturing); and (4) willingness to provide a current employee roster for the survey. Two worksites participated in our pilot research, which was conducted between September 2011 and February 2012. In addition, we recruited 20 worksites on a rolling basis between July 2012 and July 2013, all of which agreed to random assignment to condition and to complete participation in intervention and data collection activities. Following the baseline assessment, the 20 worksites were randomly assigned to intervention or delayed intervention control conditions. (See Fig 1) This paper describes findings from pilot testing the intervention in the two pilot worksites, and presents the adaptation and implementation of the OSH intervention in the 10 worksites randomly assigned to the intervention condition.

### Data collection and analysis

Data collection efforts were designed to ensure confidentiality and informed consent. For surveys, focus groups, and interviews, trained field investigators introduced themselves and the purposes of the research, and read consent information to participating employees, emphasizing that participation was voluntary and that their participation would not affect their work status. All consent was agreed upon verbally prior to completing data collection, and documented by the field investigators. Qualitative data were collected using structured interview guides, including questions and prompts developed by the investigator team; the duration of focus groups and most interviews was approximately 60 minutes. All qualitative data were collected by Dr. Pawar, MPH, BAMS (female research scientist) and other Healis field researchers (with no less than master’s degree, male and female); all were trained in qualitative data collection and in the specific scripts used in this study.

All qualitative interviews and focus groups were audio-recorded and transcribed. Additional field notes were made during the interviews and augmented later. The transcribed data were analyzed by two coders according to a conventional qualitative content analysis method [81–84], which was comprised of a two stage coding process: Level 1 structural coding and Level 2 thematic coding. Structural coding follows the structure of the interview guide, hence every question received a structural code that is applied to the appropriate text. Thematic coding was based on emergent themes that arose from review of the structural coding, and was applied in a second pass analysis. Participant quotes are included as part of the results.

#### Formative qualitative research

To assist with planning the intervention prior to the pilot intervention, in 2011, trained study staff conducted key informant interviews and focus groups to understand the local manufacturing context and setting, following defined interview scripts based on ethnographic principles [84]. The 39 key informant interviews included government officials, safety consultants, medical officers, human resource managers of worksites and personnel from industry associations, all of whom were purposively selected based on the relevant information they could provide, and recruited by telephone and face-to-face conversations. The objectives of these interviews included identifying strategies to strengthen our worksite recruitment plan, enhancing our understanding of employer attitudes toward worker health issues, and identifying strategies to engage employers around occupational safety and health. We conducted 11 focus groups at worksites, including seven with production workers (n = 86) and four with administrative/managerial staff (n = 45) in 11 companies not participating in the randomized controlled trial, based on participants’ availability. Refusals to participate were due to inconvenience or lack of availability; there were no drop-outs. Neither managers nor others were present during focus groups with workers. The purpose of these focus groups was to explore the social context of workers’ daily lives influencing their tobacco use and related environment, including relations between workers and manager, social norms, and social support; and the functional meaning of tobacco at work and the meaning of occupational health for these workers. After completion of an informed consent process, focus groups were recorded and transcribed; also, a note-taker in addition to the facilitator documented the discussions.

#### Intervention pilot study

We pilot tested several components of the intervention in two manufacturing worksites in the Greater Mumbai region, including a jewelry manufacturer (n = 346 workers) and a chemical manufacturer (n = 250 workers). This pilot tested the feasibility of the planning process and delivery of health education events for workers; in early 2012, we conducted four focus groups with workers from the two pilot worksites (2 in each) to evaluate reactions to the pilot program and garner feedback that helped inform the full intervention. At the same time, two key informant interviews were conducted (one with each of the Program Officers at each of the pilot companies) as a way to gather feedback to refine and strengthen our intervention program. Focus groups were analyzed following the methods described above; key informant interviews with managers were not formally analyzed, but the research team carefully reviewed interview notes to inform future intervention development.

#### Intervention implementation and process evaluation

We used an Intervention Tracking System during the intervention delivery. We collected information from project staff and worksite liaisons to document the extent of the implementation of the intervention (dose), the reach of the intervention (program coverage), and the fidelity of the intervention to the protocol, as well as any adaptations made to the intervention as it is delivered. Program staff completed process tracking forms at the completion of each intervention activity. Data were compiled and summarized across worksites.

#### Post-intervention qualitative data collection

Focus groups were conducted with workers (10 per group) in 2 out of the 10 intervention worksites at the completion of the intervention period, at the participating worksite. Participants included production workers who were recruited with assistance from workplace managers based on in-person discussions describing study, and following convenience sampling methods. Refusals to participate were due to inconvenience or not being available; there were no drop-outs. Neither managers nor others were present during focus groups with workers. We assessed workers’ reactions to various program components, as well as their reactions to various themes or ideas. In addition, we conducted close-out interviews with worksite managers in each of the intervention sites between late 2013 and mid-2014. Data were analyzed using standard qualitative methods to identify common themes, as described above.

## Results

### Characteristics of the sample

This paper focuses particularly on the 10 worksites included in the cluster randomized trial that were randomly assigned to the intervention condition. Of these 10 sites, one withdrew from the study before completion of the final survey [68]. The characteristics of the ten worksites randomly assigned to the intervention condition are presented in Table 1. Overall, approximately three-quarters of the workforce were in production, and men comprised 96% of the workers employed in these manufacturing companies.

**Table 1:** Characteristics of the worksites assigned to the intervention condition: Mumbai Worksite Tobacco Control Study.

Worksite	Type of business	# workers participated in survey	# workers employed based on roster	% production workers**	% male**	Multi-national (Y/N)
A*	Food & Beverages	189	203	87%	100%	Y
C	Manufacturer of Chemical products	445	480	91%	99%	Y
F	Steel processing	407	438	81%	99%	N
H	Manufacturer of industrial machinery	370	424	97%	100%	N
I	Sheet metal Fabrication, Engineering Services	135	170	56%	97%	N
L	Electricity Production	337	368	78%	90%	N
N	Petroleum	824	916	69%	92%	Y
P	Printing	266	290	67%	92%	N
R	Manufacturer of Chemical products	366	437	62%	98%	Y
T	Manufacturer of Chemical products	212	230	87%	96%	N
Total		3551	3956	76%	96%	

* Worksite “A” declined participation in the intervention.

**Percentage of production and male workers calculated as per total number of workers on roster.

### Formative research results

Formative research included key informant interviews and focus groups; central themes emerging from this research are summarized in Table 2. Through key informant interviews, we found that OSH efforts relied primarily on secondary prevention through medical screening as well as treatment of injury, rather than on primary prevention involving monitoring, evaluation, and control of hazardous exposures. Managers and workers alike reported that on-site medical care was a major component of OSH efforts; having an ambulance onsite for transporting an injured worker to the hospital, for example, was seen as an important resource. Safety officers provided further support, as mandated by the Factories Act, although in many cases, safety officers had additional responsibilities that made use of their engineering training, limiting the time available for safety issues. Key informants reported that OSH efforts relied heavily on the use of PPE. A few sites reported using engineering controls such as protective hoods for ventilation or safety switches on machines; or using administrative controls, for example, in the case of the fumes and related bad odors, job rotation was employed to limit exposure duration. Although by law, companies are to be inspected by the Directorate of Industrial Safety and Hygiene at least once a year through safety audits by factory inspectors, we saw little evidence that these inspections were conducted with regularity.

**Table 2:** Adaptation process: Core steps for translation.

*Conduct formative research to assess the local context*, *culture and existing resources*	*Summarize formative research findings*	*Adapt WellWorks intervention components based on formative research*	*Pilot test and finalize core intervention components*
Review national legislation and worksite policies related to worker safety and health Conduct focus groups and interviews with the intended audience Key informant interviews with government, safety consultants and employers – to assess employer attitudes toward worker health and strategies to engage employers around OSH Focus groups with employees – to explore social context of workers’ lives influencing tobacco use and OSH Conduct direct observations of participating worksites – to understand worksite layout, workplace flow, and places to deliver the intervention	Legislation and workplace policies The Factories Act defines maximum permissible limits for some chemicals, and requires periodic monitoring of the work environment and pre-employment and periodic screening for workers employed in enterprises using processes defined as “hazardous” under the Act. Compliance is often inadequate due to insufficient infrastructure, few trained personnel, and scarce resources devoted to inspections and enforcement. The Act requires companies to have annual safety training sessions There is also a required annual Safety Week Key Informant Interviews OSH relies considerably on secondary prevention through medical screening as well as treatment of injury, rather than primary prevention and control of hazards On-site medical care was a major component of OSH Safety officers had additional responsibilities that made use of their engineering training, limiting the time available for safety issues Considerable reliance on the use of PPE Few sites reported using engineering controls such as protective hoods for ventilation. In the case of fumes and related bad odors, job rotation was used to limit exposure duration Management expressed concern that program for workers should not impact production Focus groups with employees Working rotating and night shifts and long hour were common, and there were challenges to stay awake with frequent schedule changes Workers reported using tobacco during night shifts to stay awake, or to work faster to increase production Workers noted stressors on the job included dealing with potential hazards, such as needing to be alert when handling dangerous chemicals or dealing safely with mechanical equipment. Many workers reported that they used tobacco in response to stressors on the job Direct observations Little evidence that safety inspections were conducted regularly, even though by law worksites are required to be inspected by the Directorate of Industrial Safety and Hygiene annually No evidence of tobacco control programs	Joint worker-management participation Create worker/manager committees Build on existing health and safety committees Interventions targeting the worksite organization and environment Adopt pro-active preventive approach beyond legally proscribed standards for OSH to protect worker health Engage industrial hygienist to conduct walk-through assessments of exposure and OSH programs Provide consultations and technical assistance to management to: Reduce workers’ exposures to hazardous substances used in work process Focus on hierarchy of controls, starting with upstream strategies for materials and process substitution, engineering controls. Include recommendations for observable improvements to encourage worker engagement Emphasize importance of management commitment, employee participation, hazard analysis, hazard prevention and control, and training and education. Provide consultations to support implementation of tobacco control policies Interventions for individual workers Provide intervention activities to address both exposure to hazardous substances and health behaviors Include comprehensive program content and coordinated messages acknowledging the additive and synergistic effects of exposures to worksite hazards and individual health and safety behaviors Incorporate OSH messages into tobacco control programming and tobacco control messages into OSH programs	Joint worker-management participation Engage member of management/administration staff to assist with scheduling and promoting events and coordinate consultations with management Engage a program committee in program planning and implementation Interventions targeting the worksite organization and environment Conduct an industrial hygiene walk-through using a standardized assessment based on an adoption of a Bureau of Indian Standards checklist Based on the walkthrough, have the industrial hygienist present a brief written report to management, summarizing findings and recommending changes to reduce exposures to hazards in the work environment Provide consultation and technical assistance on the adoption, implementation, and enforcement of worksite tobacco control policies Interventions for individual workers Provide intervention activities to address both exposure to hazardous substances and health behaviors Include comprehensive program content and coordinated messages acknowledging the additive and synergistic effects of exposures to worksite hazards and individual health and safety behaviors Incorporate OSH messages into tobacco control programming tobacco control messages into OSH programs Use “success stories” to build a supportive work culture around tobacco control and OSH Provide alternative strategies to use tobacco to stay awake on the job Conduct health education sessions related to the risks associated with tobacco use and provide supports for quitting

We identified additional themes in our focus groups with workers (see Table 2). Workers reported stressors on the job, including potential hazardous exposures, needing to be alert when handling dangerous chemicals or dealing safely with mechanical equipment, and the pace of work, as described by one worker:

“I am in production, which is very much about how to have alertness, so that creates mental stress because everything should be proper, everything should be in set position, because any minor mistake or unalertness can create a bigger problem with respect to safety, because this is a chemical plant—so much more is about mental stress.”

Many workers reported that they used tobacco in response to stressors on the job. Working rotating and night shifts and long hours were common. To cope with the challenge of staying awake, workers reported using tobacco, as illustrated in the following quote:

“Some people who have night shift cannot stay awake. They have got the habit…they can stay awake only after consuming tobacco.”

### Pilot intervention results

The pilot intervention conducted in two worksites included one event for management and one for workers. Consultation for management included presentation of reports on OSH, based on the industrial hygienist walkthrough and report, and on tobacco policy, based on management interviews to assess current policies and practices. The event for workers included education around the harms associated with tobacco and occupational hazards. Company management noted the benefits of the integrated approach, as illustrated in the quote below from one of the pilot sites:

“Connecting health and safety themes together in the program ensured greater participation as it engaged users as well as non-users [of tobacco]. If the safety component had not been there, many people, especially the non-users, would have missed the event thinking that since they don’t use tobacco the event was not meant for them.”

Despite attempts to provide a balanced intervention focus, however, workers and managers alike reported in focus groups and interviews that the program emphasized tobacco control. Nonetheless, managers reported that the OSH walkthroughs were useful and provided guidance on recommended changes. Workers reported that the attention to safety demonstrated management concerns for their workers, as illustrated by the following quote:

“…Workers think that management is telling us only to complete the work…So, all this, they start thinking that, no, management is thinking all this also about us—health and safety—so some positive effect happens in their mind.”

Time and competing work demands, particularly concerns about loss of production time, were critical obstacles to participation among both workers and managers.

### Adaptation process

Following the adaptation process described above, we integrated findings from the formative research and pilot intervention to define the core components of the intervention plan. This process is summarized in Table 2. We identified and built on opportunities to utilize existing resources and standard procedures, for example, the presence of a safety officer and, depending on company size and presence of hazards, a medical officer, and existing worker training, including during safety week.

### Intervention implementation: Process evaluation and post-intervention qualitative results

Using a standard protocol, the study team delivered the intervention on a rolling basis in the ten intervention worksites over 7 to 12 months in each worksite. As noted, one of the 10 worksites randomly assigned to the intervention condition withdrew from the study and did not participate in the intervention due to a change in management in the company. We used a set of process objectives to guide the intervention and evaluate its delivery through a process evaluation system. At the completion of the intervention in each worksite, we conducted a close-out interview with the member of management or an administration staff who was appointed as the program liaison; eight of the ten worksites participated. We additionally conducted post-intervention focus groups in two randomly selected intervention worksites. We analyzed these qualitative data to identify the challenges encountered and successes achieved in our efforts to integrate OSH and tobacco control as part of the *Healthy*, *Safe*, *and Tobacco Free Worksites* program. As observed in the pilot, the interviews and focus groups confirmed that respondents perceived that the predominant focus of the intervention was on tobacco control, which was the primary motivator for management engagement in the program. In applying this model to India, we followed the WellWorks-2 structure that included three primary components; below we summarize information from both the qualitative research and process evaluation.

Joint worker–management participation: The intervention efforts in each worksite were coordinated by a member of management/administration staff who assisted with scheduling and promoting events, coordinating consultations with management, and functioning as a liaison with study staff. Following the WellWorks-2 model, we aimed to engage a program committee in program planning and implementation. Only two of the eight sites completing the close-out interview reported actively engaging a program committee, and reported that major barriers included competing responsibilities and production demands. For example, in one post-intervention interview, a manager reported that:

“The program team was not helpful. It could not be effectively formed or function because of the program team members’ own workload… Since most were production supervisors, they are too overburdened with work-related deadlines; they could not be involved in the program very much.”

Management participation in the overall intervention was inconsistent; for example, while several worksites reported no management involvement, others noted that management saw the benefits to encouraging worker engagement and facilitating tobacco policy changes.

Interventions targeting the work organization and environment: As noted in our description of the WellWorks-2 intervention, protecting worker health and safety through changes in the work organization and environment is the centerpiece of OSH interventions because primary control over occupational hazards rests with management. To adapt this approach to the Indian context, the intervention included an industrial hygiene walk-through using a standardized assessment based on an adoption of a Bureau of Indian Standards checklist [85, 86]. In our recruitment of worksites to the study, we observed that this walkthrough posed a barrier to participation for many employers who were reluctant to permit this external review of their work processes and OSH infrastructure [80]. Similarly, a manager at a participating multi-national company reported that the OSH component of the intervention was not a high priority for them, and might be a barrier for participation for similar companies more interested in the tobacco control component. We also observed that although all participating worksites employed a safety officer, the person often had additional roles and responsibilities related to the production process, posing potential competing demands.

Two industrial hygienists were trained to provide the OSH consultation, including for conducting the OSH walkthrough, preparing and presenting the report with findings, and placing the OSH component into the goals of the overall intervention. Based on the walkthrough, the industrial hygienist presented a brief written report to management, summarizing findings from the walkthrough and recommending changes to reduce potential for exposures to hazards in the work environment. Nine of the ten intervention sites participated in the walkthrough assessment. Recommendations made based on the walkthrough were related to housekeeping, ventilation, making simple and doable changes in the physical infrastructure (e.g. marking of areas, cementing floors), use of PPE, improvements in engineering controls and shielding open machinery parts. Among the Indian-owned companies, five of the six reported making some changes in response to the report; although changes made generally required few resources (e.g., additional signage around hazardous areas, housekeeping), one company reported exploring designating a dedicated safety officer as a resulted of the intervention. Three of the eight companies participating in the close-out interviews reported having “strong” OSH programs already in place, either in response to requirements associated with the nature of their industry (e.g., oil and natural gas) or because they were part of a multinational corporation that required compliance with stringent corporate-wide OSH standards and found further input unnecessary. In follow up focus group discussions in two worksites, workers consistently reported that they were not aware of any changes to improve the work environment.

We also provided consultation related to the company’s tobacco control policy, including consultation and technical assistance on the adoption, implementation, and enforcement of worksite tobacco control policies. Six of the ten worksites already had tobacco control policies in place to restrict or ban smoking or tobacco use on site due to fire hazards or quality control concerns; by the end of the intervention, all nine sites participating in the intervention had a policy, including six of the nine that had written policies in place. For example, one manager reported that:

“The management easily accepted the idea of adopting a tobacco policy mainly especially because it did not involve any costs to the company.”

Other companies reported that the tobacco policy and accompanying signage provided an “important display for visitors,” and key information for new recruits “to know that these habits are not permitted at the workplace.”

Interventions for individual workers: Most participating worksites reported having annual safety training as part of compliance with the Factories Act, and reported that the content of these training sessions included, for example, mock fire drills and use of PPE. All of the companies also reported that they observed the required annual safety week. None of the participating companies had prior tobacco control programs to promote or support tobacco use cessation.

We were able to implement the worker-level program fully in seven and partially in two of the 10 intervention worksites. The program included six health education events at the worksite for workers, regardless of whether they used tobacco or not. Conducted by trained health educators from the study, each of the six events was offered for one day in each company, allowing maximum participation by workers, including workers on evening and night shifts. Each event was delivered on a single day during multiple 15 to 20 minute sessions at each intervention company, and was open to all workers. The purpose of these visits was to enhance workers’ understanding of the risks associated with tobacco use, increase their motivation to quit or help someone quit, and build the skills and social support needed for cessation. The messages and materials additionally linked tobacco control with the work environment. By including those who did not use tobacco, we aimed to expand messages around a safe and healthy work environment, and to provide supports for quitting and build social norms around tobacco control. To accommodate management concerns about timing and production impacts, the programming was generally scheduled during work breaks or at the end of shifts to prevent disruptions to production. Some workers reported that time pressures, workload and the need to meet production targets constrained their participation in the program; directives from supervisors to attend the program contributed to increased participation.

We aimed to use “success stories” to build a supportive work culture around tobacco control and OSH. Although we were able to highlight successes around quitting tobacco and supporting others in their quit attempts, managers were generally not willing to highlight OSH efforts in a similar way; we were only able to incorporate an OSH success story in one of the 10 intervention worksites. Employers were generally unwilling to open discussions of OSH-related issues either with workers or study staff. Not surprisingly, in focus group discussions with workers, we learned that most workers thought the program was about quitting tobacco, which was the main study outcome, and most had little awareness of the minimal OSH efforts.

We also developed messages around tobacco control that took into account the conditions of work. For example, we learned from our formative research that rotating shifts were common, and workers reported challenges trying to stay awake with the frequent schedule changes, as noted above. Workers reported using tobacco during night shifts to stay awake, or to work faster to increase production. We incorporated messages about alternative strategies to address these potential functions of tobacco use. Workers also noted that stressors on the job included dealing with potential hazards, such as needing to be alert when handling dangerous chemicals or dealing safely with mechanical equipment. Many workers reported that they used tobacco in response to stressors on the job. Although we were able to incorporate stress reduction into the intervention messages, we were not able to effect changes in work practices to address organizational sources of stress.

Additionally, themes identified from the key informant interviews with management and company liaisons also highlighted several adaptations made in response to the local setting:

- Building on existing practices: Although in many companies there was little tradition of health promotion programming, we were able to build on other common practices. For example, in one company, management representatives noted that:

“Since the company already has a concept of daily ‘Tool Box Talks’ with employees every morning, this platform was effectively used for conducting the health education sessions.”

Similarly, another company reported that “the company has a protocol of conducting morning assembly before the workers begin the day’s work; the sessions were conducted during that time.”

- Consideration of the cultural context. For example, in one company, the educational program was conducted in the canteen because the health educators were not allowed in the plant. However, this posed a barrier for some workers. As a manager in this company reported:

“Women workers don’t often go to the canteen, hence they missed out. But the message did reach them through posters and banners. Separate events could have been conducted for the ladies. Pamphlets could have been circulated to each worker even if the event could not reach all.”

- Variations in program delivery. Flexibility in the delivery of the program was cited repeatedly as important to managers, including flexibility in scheduling, number of sessions, and ability to locate the program in multiple locations due to the large size of the plants. For example, one manager reported:

“It was difficult for your staff to cover the area of the company; we could not always provide vehicles due to their unavailability. And it was difficult to gather employees at one place due to time constraints, so sessions had to be conducted in individual locations many times. So, one program event should have been conducted at least thrice to cover the maximum employees.”

## Discussion

The health of workers is of paramount importance, not only for workers and their families but also to ensure the competitive advantage and productivity of employers, with clear implications for national economies [15, 17]. In light of the rising global impact of NCDs as well as the persistent impact of occupational hazards on worker health, we examined the feasibility of adapting and implementing an integrated worksite intervention addressing tobacco control and OSH hazards in India. This study illustrates a systematic process for adapting an evidence-based intervention, tested initially in the U.S., to a LMIC. This intervention resulted in significant improvements in tobacco use cessation among production workers [68]. We encountered challenges, however, in fully implementing the OSH intervention, reflecting our experiences in engaging participating worksites as well as obstacles in the broader environment.

This integrated approach to worker safety and health was new to participating companies. The intervention relied on engaging a member of management to serve as a liaison in coordinating the program in the worksites, and on a program committee to assist with planning an implementation. Although all sites appointed a program liaison, only two sites were able to appoint and benefit from the work of a program committee. Production demands and competing work priorities limited participation of the program committees, and similarly placed constraints on the level of involvement of the program liaisons. The approach to OSH in general differed considerably from that observed in our U.S.-based work; for example, the OSH infrastructure relied predominantly on secondary prevention (i.e., medical screening) and the treatment of injuries, rather than primary prevention and control of hazards, and there was little experience with worker-management committees. We also observed generally low levels of leadership support and commitment to OSH, evidenced by minimal management participation in the intervention, reluctance to discuss OSH issues with the study team or workers, and little receptivity to recommendations resulting from the industrial hygienist’s reports. As we have seen in our research in the U.S., there was a tendency to gravitate toward interventions targeting individual behavior change, here focused on tobacco control, rather than on efforts to reduce workplace-wide exposures to occupational hazards. While it is important to consider the needs of specific worksite settings, our experiences also highlight the importance of considering broader national and global trends that shape workers’ experiences on the job, their related health outcomes, and the influence of worksite interventions.

Globalization plays a complex role in shaping the OSH resources and experiences of LMIC’s. With economic globalization comes increasing movement of goods, services, and technology across international borders [87, 88]. These global supply chains include foreign direct investments by multinational enterprises, often creating new opportunities for employment within LMICs. When these employers adhere to international labor standards and OSH regulations, these investments may contribute to opportunities for “decent work” in safe work environments [89]. Global supply chains may also, however, perpetuate already-existing threats to OSH within LMICs. For example, as a result of outsourcing of jobs within the supply chain, the lead enterprise may not be responsible for employment of those producing the goods or services, and therefore not take responsibility for their working conditions [87, 89]. Competition in global commerce pressures employers to structure work as efficiently as possible, creating stress in the work organization, scheduling and staffing that likely increase risks for worker illness and injury [90]. In addition, subcontractors in the global supply chains may cope with competition pressures through the use of forms of employment and work environments that may not comply with international standards. These cross-border supply chains pose challenges for compliance with OSH legislation at the national level; in addition, many LMIC’s are unprepared to monitor compliance across workplaces [89].

The challenges encountered in implementing the OSH component of this intervention illustrate the impact of globalization. Despite leadership provided by Indian organizations such as the National Institute of Occupational Health (NIOH), Industrial Toxicology Research Center (ITRC), and the Central Labour Institute, large gaps remain in occupational health and hazard surveillance and in understanding the complexity of issues in the Indian setting [91, 92]. Although legislation is in place to provide protections to workers in the formal sector, these laws appear to have little teeth because there are inadequate resources devoted to enforcement, few trained professionals to conduct inspections or institute protections within workplaces, little incentive for employers to invest in worker health and safety, and few norms within the business community that might foster the benefits of a “good place to work” [93]. In terms of human capital alone, Pingle reported that in 2012, there was a need for about 10,000 occupational health physicians and industrial hygienists just for the organized sector, but in 2012, they only numbered 1,000 [93]. We observed the lack of trained personnel in this study; few trained industrial hygienists were available to work with us, and safety officers employed by worksites were often trained as engineers and had significant production-related responsibilities beyond safety. Indeed, there is increasing recognition of the need for an international response to provide professional education and training to build a cadre of trained OSH professionals in LMICs [94]. Industrial hygiene strategies used in industrialized nations, relying on identification and control or elimination of hazards as a primary strategy for prevention, are often resisted in India because of the costs of compliance and labor market forces [92, 95]. In most cases, where occupational health efforts do exist, they are most evident in medical services attached to workplaces that offer general medical diagnosis and treatment, with little if any attention to prevention and protection [21, 96]; these reports align with our observations in this study.

Across employers, there are significant disparities in available resources. As is also true for the U.S. [97], the consequences are not evenly distributed across society: workers with few skills, less education, and in lower social positions are at elevated risk for encountering hazardous conditions of work, including physical exposures, high noise levels, ergonomic risks, shift work, a hectic work place, and lack of job control [3, 20, 98]. Increasing demands for productivity may increase pressures on workers [99], observed here in worker reports of rotating and night shifts and long hours in the context of 24-hour production schedules. Despite the few cases in which globalization may actually contribute to reductions in hazardous exposures, for example through investments in new workplaces with safer technologies or importing internationally supported standards of OSH through multinational corporations [16], we observed that OSH was generally given a low priority in the face of production demands.

As an additional challenge, India has little tradition of employer-sponsored wellness or health promotion efforts, although interest in these areas is emerging [100–103], under the leadership of key multinational corporations [104, 105]. Several reports of studies of workplace interventions to reduce NCD risk in India have demonstrated feasibility [100] and efficacy [103]. These discussions have generally focused on wellness to the exclusion of OSH [106], providing little foundation for building the OSH infrastructure.

Enterprises may decide to implement solutions to protect and promote worker health for multiple reasons [99]. From an ethical perspective, it is the “right thing to do”[22]. For example, the United Nations Global Compact encourages corporations to adopt sustainable and socially responsible policies and support fundamental principles for human rights [107]. In some settings, incentives may be provided through requirements around corporate social responsibility [99], although in India, OSH and welfare activities for workers cannot be legally considered to meet requirements for corporate social responsibility. Much manufacturing in LMICs, however, is based on investment from foreign firms seeking lower production costs resulting from low wages and limited health and environmental infrastructures within a global supply chain that depends on labor standards below that for comparable workers in higher-income countries [20]. A starting point for OSH efforts relies on comprehensive legal standards. With global efforts by organizations such as WHO and the International Labor Organization (ILO), most countries have legislation requiring minimum standards for worker protection [16, 17, 22, 108]. Despite safeguards, however, in response to the new trade agenda there has been strong opposition to uniform OSH standards, and reductions in support for OSH and labor standards in support of business and trade interests [17, 20]. Similar responses to national tobacco control policies have been waged by the U.S. Chamber of Commerce and others advocating for business interests over the interest of public health [109].

Nonetheless, legislation can make a difference in worker health outcomes. For example, in the U.S., the Occupational Safety and Health Administration of the U.S. Department of Labor (OSHA) rules have resulted in reduced exposures and illnesses [110], and reductions in injuries in workplaces in which there have been inspections and enforcement [111, 112]. Recognizing the complexity of OSH problems, additional recommendations have been made for improvements in the structure of OSH protections in the U.S., which may have implications for legislation in LMIC’s. For example, requiring employers to certify that their workplaces have passed an annual inspection for OSH regulatory compliance may help to reduce fraud and conflicts of interest, and further benefits may be gained by mandating safety and health management systems that effectively find and fix recognized hazards, or by establishing rights for individual workers to take legal action for relief from workplace hazards [113].

Others have articulated a business argument focused on the benefits of keeping workers healthy and safe [114, 115]. This framing may be incorporated into efforts to link working conditions with other management goals [22, 116, 117]. A recent survey found that the salient factors in making the business case for employee health may vary significantly across countries depending on cost drivers [118]. For example, this survey showed that among U.S. companies, the number one reason for adopting wellness programs for employees was to manage health care or insurance costs, whereas in all other parts of the globe, major reasons included improvements in productivity and performance, worker health or morale, and decreases in absenteeism and presenteeism. In our experiences with the Mumbai study, employers expressed major concerns with productivity and absenteeism, and required assurances that programming would not disrupt production activities or schedules. Managers reported that tobacco control efforts provided advantages for production by reducing break times to use tobacco, addressing concerns about maintenance costs related to spitting tobacco or discarded tobacco packages, reducing fire hazards, and reducing potential for product contamination that might result when tobacco was used while working. Future research is needed that integrates the business case for wellness initiatives with that for OSH efforts, and that can help to frame OSH as an investment with benefits for productivity and economic security rather than a luxury [99].

Future research is also needed to identify incentives and drivers to encourage broader implementation of effective protection and promotion of worker health and safety in LMIC’s. One area of promise is the possible role for social impact bonds, an innovative financing tool that uses up-front funding from private or philanthropic investors to promote evidence-based programs [119, 120]. In addition, further research may help to identify strategies to leverage the leadership roles of vanguard companies, such as multinational corporations or other large companies, who may help to set new standards for business practice. There is also a significant need for research to identify effective interventions for the large majority of workers in LMICs working in the informal sector. Such interventions may additionally address community and social resources that can intersect with the informal economy. Finally, cross-national research may help to identify effective policies and strategies to counter the health threats arising from the “race to the bottom” in the global economy.

We acknowledge several limitations to the case study presented here. We were not able to observe and directly measure the extent to which employers made changes to reduce the potential for exposure to occupational hazards as part of this study, although as we have reported here, our process tracking data indicate that few changes were made. Although we have included observations about the multinational corporations included in this study, the sample size is too small to allow us to draw reliable inferences about differences based on status as a multinational corporation. We recognize that the legal, economic, social, and political context of work exposures, policies, and practices differ by country and the effects of globalization may vary among countries and regions: India represents only one case example. As we have noted, our focus on the formal sector does not reflect the experiences of a large portion of workers in India who work in the informal sector. Further, our work was in one region and in one industry sector in a large, diverse country. While we believe that our overall conclusions are sound, our specific observations are not necessarily generalizable to India overall or elsewhere.

## Conclusions

Any attempts to export approaches to worker health, safety and wellbeing will need to rely on effective adaptation of evidence-based methods, taking into account the complex political, legal, social and economic forces that clearly shape their implementation. Research is needed to expand understanding of factors influencing the adaptation process within the resource constraints typical of LMICs [67]. An exploration of the social and political forces that contribute to global OSH disparities has been initiated by others [121, 122]; more attention to this concern is clearly warranted, and we believe this study contributes to this ongoing discussion. Implementation research is also needed to explore the complexities and challenges of intervening to improve the work organization and environment. Critical avenues of inquiry include the role of the external environment in shaping effective implementation within enterprises, including the influence of national legislation, policies and incentive structures, the role of vanguard organizations in establishing competitive pressures to protect and promote worker health, and effect of networks of employers in fostering supportive collective practices [123]. These adaptation and implementation processes also involve reciprocal relationships, recognizing the contributions from LMICs, such as strategies to provide rapid responses to the short-term needs [12, 124] and novel solutions to threats to worker health, potentially with improved affordability and simplicity [12, 125, 126].

In conclusion, India presents one example of the changing influences on worker health in LMICs, including the rising burden of NCDs and the intensified exposures to both physical and organizational hazards on the job, compounded by increasing social disparities. As increasing attention is given to the rising burden of NCDs in the developing world, it is critical that national policies and enterprise initiatives go beyond a singular focus on individual health behaviors to incorporate improvements in working conditions that clearly shape health outcomes, particularly for the most vulnerable workers.

## Supporting information

S1 File COREQ checklist. (PDF)
